# Serum biomarkers confirming stable remission in inflammatory bowel disease

**DOI:** 10.1038/s41598-021-86251-w

**Published:** 2021-03-23

**Authors:** Christoph Kessel, Miha Lavric, Toni Weinhage, Markus Brueckner, Sytze de Roock, Jan Däbritz, Jakob Weber, Sebastiaan J. Vastert, Dirk Foell

**Affiliations:** 1 Pediatric Rheumatology and Immunology, University Hospital Muenster, Domagkstr. 3, 48149 Muenster, Germany; 2 Research Group Ambient Intelligence, Saxion University of Applied Sciences, Enschede, The Netherlands; 3 Department of Gastroenterology and Hepatology, University Hospital Muenster, Muenster, Germany; 4 Department of Pediatric Rheumatology and Immunology and Center for Translational Immunology, University Medical Center Utrecht, Utrecht, The Netherlands; 5 Department of Pediatrics, University Medical Center Rostock, Rostock, Germany; 6 BÜHLMANN Laboratories AG, Schoenenbuch, Switzerland

**Keywords:** Inflammatory bowel disease, Biomarkers, Prognostic markers

## Abstract

Crohn's disease (CD) and ulcerative colitis (UC) have a chronic-remittent course. Optimal management of inflammatory bowel diseases (IBD) relies on early intervention, treat-to-target strategies and a tight disease control. However, it is challenging to assess the risk of relapses in individual patients. We investigated blood-based biomarkers for the confirmation of disease remission in patients with IBD. We retrospectively analyzed samples of 40 IBD patients (30 UC, 10 CD) enrolled in a tight-control follow-up study. Half of the patients had a flare during follow up. Serum was analyzed for S100A12 as well as S100A8/A9 and for 50 further biomarkers in a bead-based multiplex assay. The concentrations of 9 cytokines/chemokines and S100A8/A9 significantly differed in IBD patients with unstable remission (before flares) when compared to IBD patients with stable remission. Although the number of patients was small, ROC curve analyses revealed a number of biomarkers (IL-1β, IL-1RA, IL-8, IL13, IL-15, IL-21, IL-25, IFN-β, CXCL9, CXCL10, CXCL11, Galectin-1, G-CSF and S100A8/A9) that were elevated in patients with later occurring relapses. While earlier studies on peripheral biomarkers in IBD are limited to only few analytes, our study using a broad screening approach identified serum biomarkers with the potential to indicate unstable disease control in IBD, which may help to steer individual therapies to maintain remission.

## Introduction

The management of patients with inflammatory bowel disease (IBD) is evolving. The traditional concept of a step-up therapy has been challenged and treat-to-target strategies have been proposed^1^. The main treatment target is to induce and maintain disease remission, which means a control of intestinal inflammation, a normalization of life, and the prevention of long-term damage^2–5^. Both for Crohn’s disease (CD) and ulcerative colitis (UC), effective biological drugs enable improved therapeutic outcomes^6^. Disease remission can be defined by endoscopic endpoints such as mucosal healing. Yet, radiologic status, patient reported outcomes, and use of non-invasive biomarkers are also conceivable measures of the therapeutic target^7,8^.

Follow-up recommendations for patients with IBD based on treat-to-target strategies mainly focus on the initial treatment phase, when therapies are started in patients with active disease to induce remission^4^. However, patient follow-up upon successful initial treatment is less clear. It is important to maintain a sustained remission. Since CD and UC are both chronic-remittent diseases, quiescent phases may be followed by (seemingly unprovoked) relapsing disease. Therefore, monitoring of disease activity is the mainstay of clinical decision-making. At present, accurate monitoring of intestinal inflammation relies upon clinical indices (based upon symptoms and clinical examination) and endoscopy, in conjunction with histological investigation and imaging techniques. However, these diagnostic options have a number of drawbacks, as they are time consuming, costly, invasive and/or not necessarily objective. Indirect, yet reliable, measures of biological disease activity are of utmost importance. Blood tests, including C-reactive protein (CRP) and erythrocyte sedimentation rate (ESR), are in common use but have insufficient sensitivity and specificity for intestinal inflammation^9^.

Currently there are no means to predict the long-term disease course, and adjusting treatment to the actual needs of patients is especially difficult when the patient is feeling well. In these phases, invasive measures of subclinical disease activity such as endoscopy are often not considered acceptable^10^. Objective measures by biomarkers would thus be helpful in evaluating the risk for relapses^11–13^. Even though some biomarkers measured in blood or stool have been shown to offer variable degree of utility in monitoring gastrointestinal tract inflammation in IBD, in clinical practice there is still an unmet need for biomarkers that could assess the stability of disease remission and the risk of relapse^14–17^. The effect of tight control management on Crohn’s disease (CALM) trial has demonstrated that treatment escalation based on symptoms combined with elevated serum CRP and/or fecal calprotectin was better than symptom-based escalation alone^18^. Despite a robust diagnostic accuracy, the use of fecal markers is somewhat difficult in everyday practice especially from the patients’ perspective^19,20^. Consequently, stool markers are actually monitored in a minority of patients^21,22^. Patients with IBD prefer blood-based over fecal biomarkers^23^. However, blood-based bio-markers have shown poor accuracy^24^. A need therefore exists for blood-based biomarkers that accurately detect disease activity in IBD.

In a previously published prospective 36-month multicenter study, we demonstrated the utility of fecal biomarkers to predict the flare risk in IBD patients after reaching inactive disease^25,26^. Time course analysis of S100A12 up to 9 months before and after relapse showed a clear increase of fecal but not serum S100A12 concentrations up to 6 months before clinical relapse. We now extend our work to candidate biomarkers that appear potentially related to inflammatory processes in IBD and can be analyzed in serum samples collected during remission. We aimed at identifying biomarker panels to identify patients who may need an optimized and/or intensified maintenance therapy to avoid disease flares.

## Materials and methods

### Patients and study design

In a prospective multicenter study, patients with IBD in remission were consecutively recruited and followed up between April 2008 and June 2011 in four independent German outpatient specialized clinics as previously described^25,26^. The diagnoses of CD and UC were confirmed as described previously^25,26^. Patients with coexisting and serious cardiopulmonary, hepatic, renal, neurologic, psychiatric, and rheumatologic disease, a history of HIV and/or hepatitis B and C were excluded from the study. Patients were assessed at a minimum of 3-month intervals or when relapse occurred. Serum and stool samples were prospectively collected at each visit when available. In addition to baseline characteristics, symptoms, medication, clinical signs, and standard laboratory results (full blood count, ESR, CRP) were recorded throughout the study. For the present analyses, 80 serum samples were retrospectively selected from 40 IBD patients based on the occurrence of disease flares at follow-up within a maximum of 1 year. For each patient paired samples either during an initial (visit 1) or a follow-up visit (visit 2) were available, making up 60 samples from 30 UC patients and 20 samples from 10 CD (Table 1). Half of patients in both groups either remained in stable remission (experiencing remission both at visit 1 and 2) or were classified as unstable remission (with remission at visit 1, but an acute flare during follow-up visit 2). Among CD patients, five were classified as stable and five as unstable remission. Baseline characteristics are summarized in Table 1. The study was approved by the Ethics Committee of the University of Münster (ref. no. 2006-267-f-S), and written informed consent was obtained from all patients*.* The authors confirm that all experiments were performed in accordance with relevant guidelines and regulations.

**Table 1:** Characteristics of included IBD patients.

	UC		CD	
	Stable	Unstable	Stable	Unstable
Patients (n)	15	15	5	5
Age at visit 1 (years, median; range)	52.6; 27.9–69.8	46.6; 20.8–70.2	32.4; 27.5–55.3	30.5; 19.4–47.0
Gender (male/female)	7/8	5/10	1/4	3/2
Disease duration (years, median; range)	11.8; 0.2–31.0	16.1; 1.7–29.7	23.4; 4.4–28.5	16.4; 3.0–22.6
BMI at visit 1 (kg/m^2^, median; range)	27.5; 20.2–36.0	27.4; 19.8–32.4	25.2; 18.1–28.1	20.4; 18.9–30.1
UCAI at visit 1 (median; range)	1; 0–3	1; 0–3	–	–
CDAI at visit 1 (median; range)	–	–	40; 10–143	16; 10–147
Days from visit 1 to 2 (median; range)	74; 27–308	31; 23–77	97; 17–275	74; 51–133
**Therapy (n)**				
Steroids (systemic)	0	0	1	1
Steroids (local)	1	0	1	0
Azathioprine	2	0	0	1
Mesalazine	4	0	1	0
Anti-TNF	0	0	2	2
**Localization (n)**				
Colonic	–	–	1	1
Ileocolonic	–	–	4	4
Ulcerative proctitis	2	1	–	–
Left-sided colitis	11	8	–	–
Pancolitis	2	6	–	–
**Routine laboratory markers**				
Hb (g/dl, median; range)	13.7; 11.5–16.5	13.9; 11.2–15.7	13.3; 11.7–15.6	13.2; 11.6–13.2
WBC (10^3^/µl, median; range), P	6.75; 4.45–9.55	7.12; 4.52–12.8	6.89; 5.13–9.73	6.97; 5.41–11.08
CRP (mg/dl, median; range)	0.3; 0.3–0.9	0.7; 0.2–3.0	0.4; 0.3–0.7	0.3; 0.3–0.9

*IBD* inflammatory bowel disease, *UC* ulcerative colitis, *CD* Crohn’s disease, *TNF* tumor necrosis factor, *Hb* hemoglobulin, *WBC* white blood cells, *CRP* C-reactive protein*.*

### Assessment of disease activity

Disease activity was assessed based on the Crohn’s disease activity index (CDAI) for patients with CD and the ulcerative colitis activity index (UCAI) for patients with UC. Remission was defined as a CDAI < 150 or UCAI < 5. Relapse was defined as follows: CDAI > 250 over 2 consecutive weeks or a CDAI > 150 with an at least 70-points of increase within 2 weeks as compared with CDAI at the previous study visit; UCAI > 6 over 2 consecutive weeks or a UCAI > 4 with an at least 3-points of increase within 2 weeks as compared with UCAI at the previous study visit.

### Immunoassays

Concentrations of S100A12 were determined by a double-sandwich ELISA, as described previously^27,28^. Calprotectin (S100A8/A9) was measured by a commercial sandwich ELISA (Bühlmann Laboratories AG, Schoenenbuch, Switzerland). Validated multiplexed immunoassays were used to measure 50 analytes using Luminex xMAP proteomics technology (Austin TX, USA). Fifty different carboxylated magnetic beads, each with a distinct emitting fluorescence pattern, were purchased from Luminex Corporation (Austin, TX, USA). Capture antibodies (commercially purchased) for 50 analytes were covalently coupled to the microspheres as described previously^29–31^. Acquisition was performed with a BioRad FlexMAP3D (BioRad laboratories, Hercules, USA) in combination with xPONENT software, version 4.2 (Luminex). Data were analyzed by 5-parametric curve fitting using Bio-Plex Manager software, version 6.1.1 (BioRad).

### Statistical analysis

Results were analyzed using GraphPad Prism 8.0 and R 3.5.0 (The R Foundation for Statistical Computing, Vienna, Austria) and Statistical Package for the Social Sciences (SPSS version 26, IBM New York, USA). For pairwise comparison of serum analyte levels between remission and acute flare phases, Wilcoxon signed-rank test was used. For comparing results between groups, Mann–Whitney *U* test was used. Kruskal–Wallis test with Bonferroni post-hoc analyses were applied to correct for multiple comparisons. Inferential statistics were intended to be exploratory, not confirmatory, and were interpreted accordingly. Receiver operated characteristics (ROC) curve analyses (GraphPad Prism 8.0) were applied to test for the prediction of flares and the distinction of populations at risk (stable or unstable remission groups), with calculation of the area under curve (AUC). Binary logistic regression analyses were performed to test for multiparametric prediction models. The significance level was set at P < 0.05 and confidence levels at 95%.

## Results

**Figure 1:**
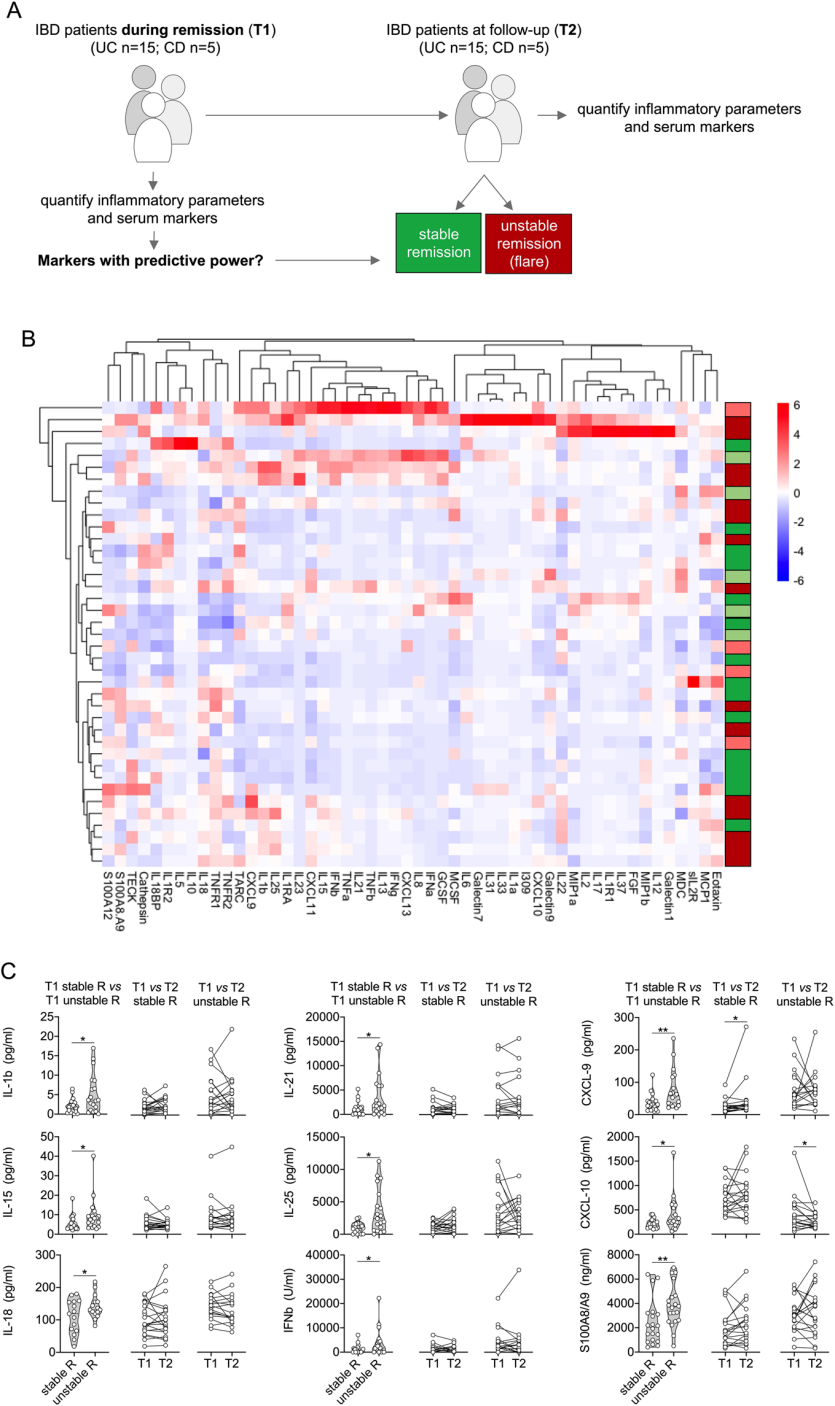
Analysis of inflammatory parameters and multiplexed serum markers in IBD. (**A**) Illustration of the experimental layout. IBD patients during remission were included into the study (T1). Patients were followed-up over 1 year. Half of the patients had a relapse during follow-up. A second sample was obtained at T2, either at the time of the flare of at the end of follow-up in remission. (**B**) Heatmap of serum marker data from bead array assay, ELISA data (S100A8/A9, S100A12) and routine inflammatory parameters (ESR, CRP, WBC) following unsupervised hierarchical clustering and complete linkage analyses (RStudio R 3.5.0, the R Foundation for Statistical Computing, Vienna, Austria). Red and green indicate future relapse or stable remission of UC patients, light red and light green indicate future relapse or stable remission of CD patients. (**C**) Markers with significantly different levels at T1 between IBD patients with stable remission (stable R) or unstable remission (unstable R) (left panel column), and comparison of respective marker levels between T1 and T2 during stable (middle panel column) or unstable remission (right panel column). Acquisition was performed with a BioRad FlexMAP3D (BioRad laboratories, Hercules, USA) in combination with xPONENT software, version 4.2 (Luminex). Data were analyzed using Bio-Plex Manager software, version 6.1.1 (BioRad). Data of individual serum biomarkers were analyzed by Mann–Whitney *U* or, when paired, by Wilcoxon signed rank test. *p < 0.05, **p < 0.01.

**Figure 2:**
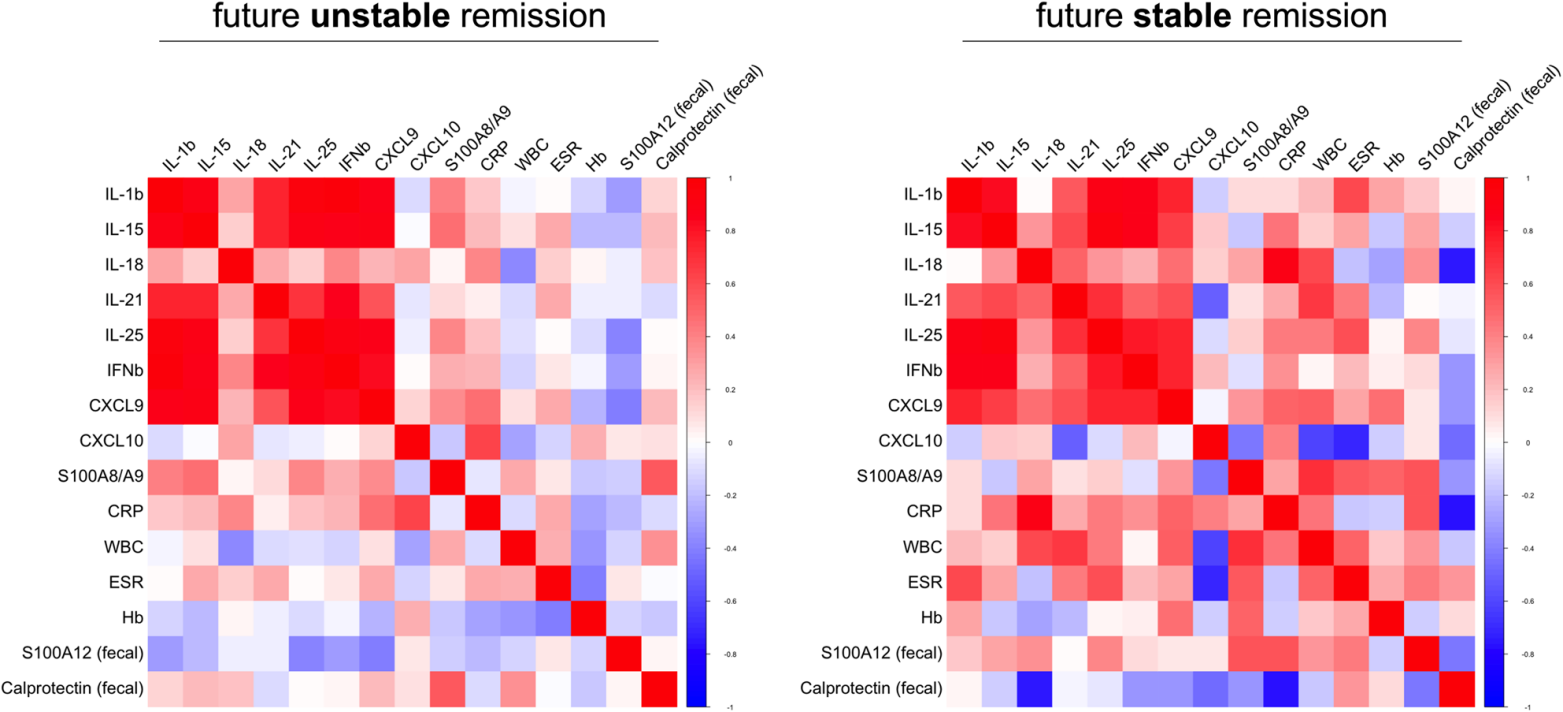
Multiple correlation analyses of inflammation biomarkers in IBD. Serum markers with significantly different levels at T1 based on whether experiencing future flare or remaining in remission (as in Fig. 1C) as well as routine clinical laboratory (WBC, ESR, CRP) and fecal markers of inflammation were analyzed for their association based on spearman rank (RStudio, R 3.5.0, the R Foundation for Statistical Computing, Vienna, Austria).

**Figure 3:**
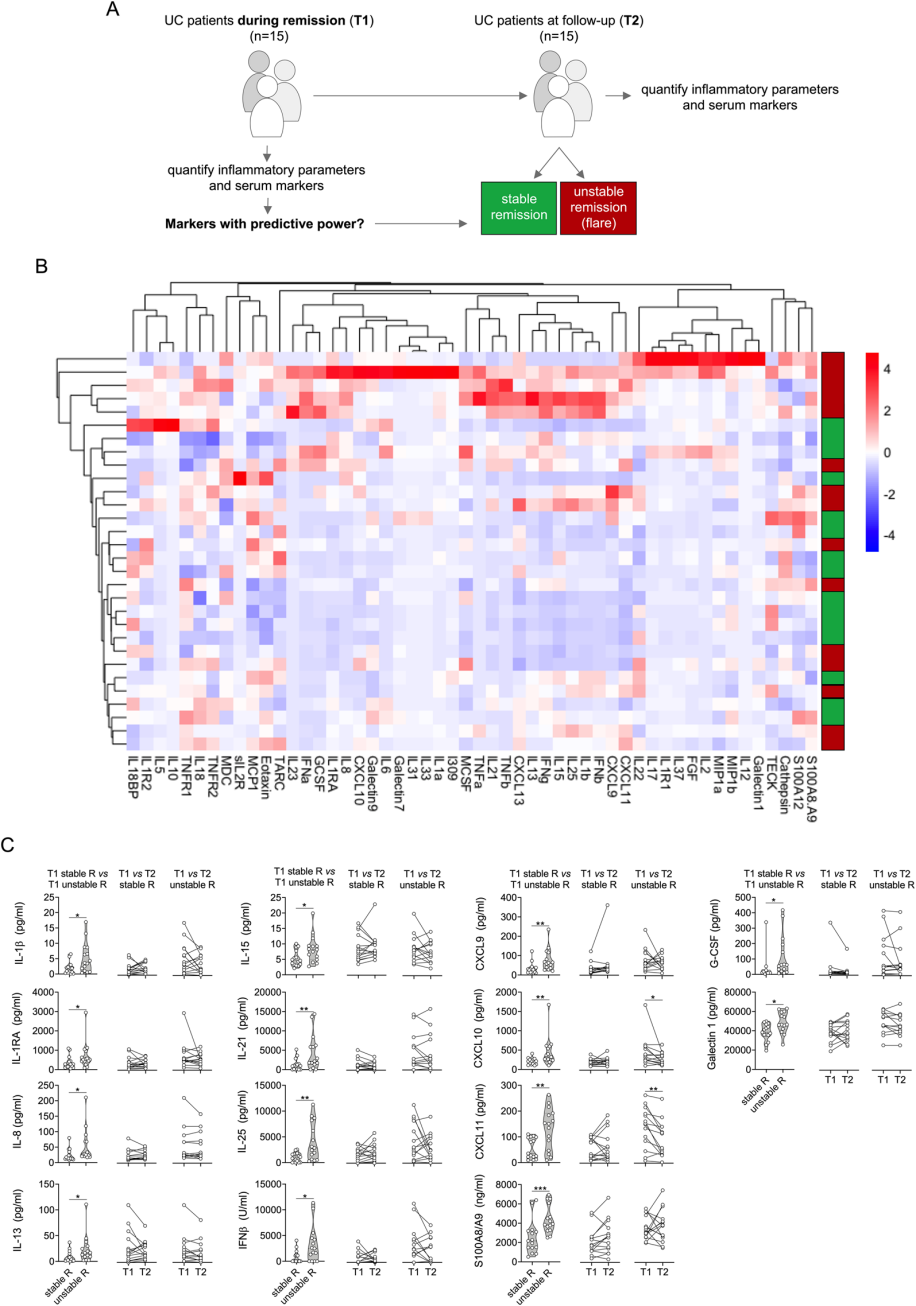
Analysis of inflammatory parameters and multiplexed serum markers in UC. (**A**) Illustration of the experimental layout, restricted to UC patients during remission at inclusion (T1). Patients were followed-up over 1 year. Half of the patients had a relapse during follow-up. A second sample was obtained at T2, either at the time of the flare of at the end of follow-up in remission. (**B**) Heatmap of serum marker data from bead array assay, ELISA data (S100A8/A9, S100A12) and routine inflammatory parameters (ESR, CRP, WBC) following unsupervised hierarchical clustering and complete linkage analyses (RStudio, R 3.5.0, the R Foundation for Statistical Computing, Vienna, Austria). (**C**) Markers with significantly different levels at T1 between IBD patients with stable remission (stable R) or unstable remission (unstable R) (left panel column), and comparison of respective marker levels between T1 and T2 during stable (middle panel column) or unstable remission (right panel column). Acquisition was performed with a BioRad FlexMAP3D (BioRad laboratories, Hercules, USA) in combination with xPONENT software, version 4.2 (Luminex). Data were analyzed using Bio-Plex Manager software, version 6.1.1 (BioRad). Data of individual serum biomarkers were analyzed by Mann–Whitney *U* or, when paired, by Wilcoxon signed rank test. *p < 0.05, **p < 0.01, ***p < 0.001.

**Figure 4:**
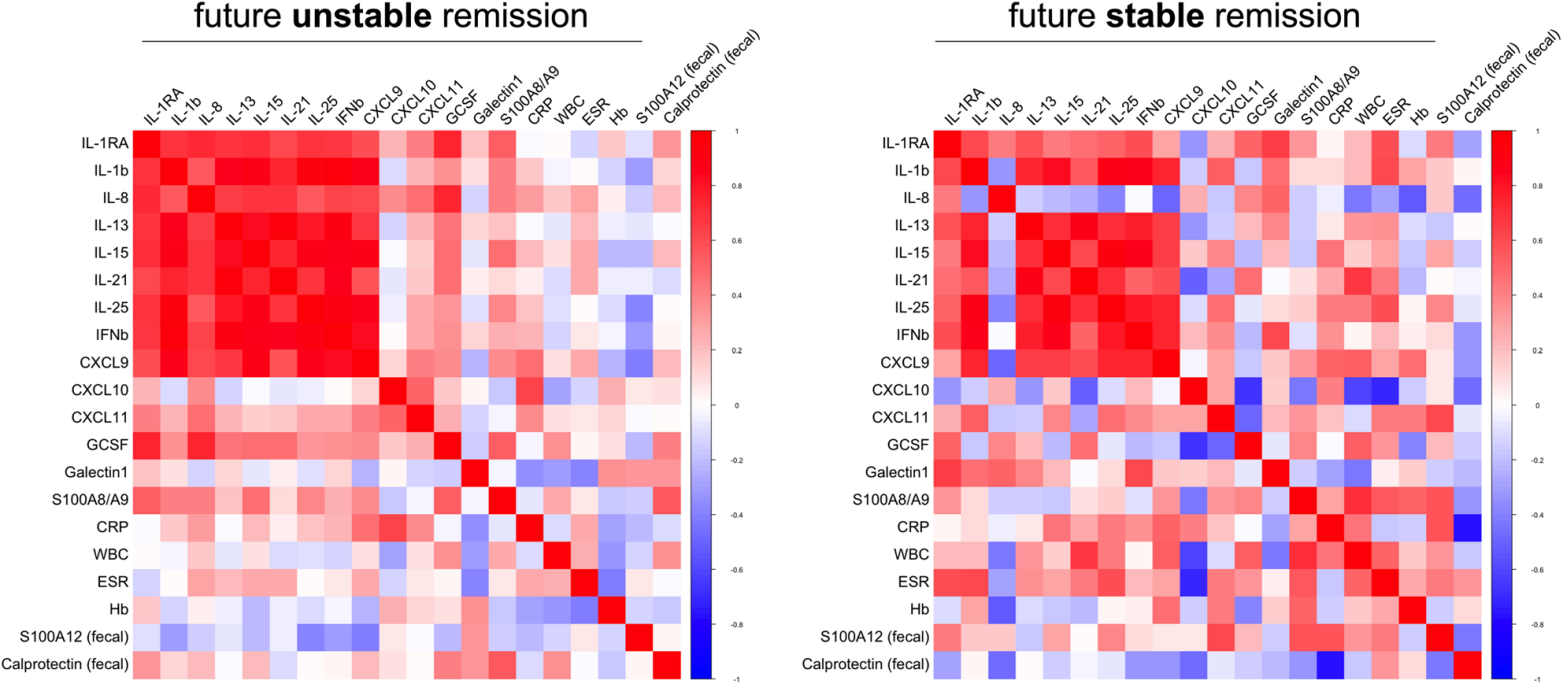
Multiple correlation analyses of inflammation biomarkers in UC. Serum markers with significantly different levels at T1 based on whether experiencing future flare or remaining in remission (as in Fig. 3C) as well as routine clinical laboratory (WBC, ESR, CRP) and fecal markers of inflammation were analyzed for their association based on spearman rank (RStudio, R 3.5.0, the R Foundation for Statistical Computing, Vienna, Austria).

Our broad serum biomarker analyses in IBD followed a specific methodology: a first sample was available initially when the patients were recruited, and all patients were in disease remission at this time point (Table 1). During the followed-up period over 1 year, half of the patients had a relapse. A second sample was obtained at T2, either at the time of the flare or at the end of follow-up in remission (Figs. 1A and 3A). Stable remission and unstable remission datasets were compared between each other for the whole cohort (Figs. 1 and 2), followed by independent separate analyses for CD and UC (Figs. 3 and 4).

Within the IBD cohort, the acquired serum level data of 52 analytes did not result in specific grouping of patients when subjected to unsupervised clustering analyses (Fig. 1B), but yielded 9 markers with concentrations that differed in patients with future unstable remission compared to those with future stable remission (i.e. without subsequent relapse): already at the baseline visit (T1) serum levels of IL-1β, IL-15, IL-18, IL-21, IL-25, IFN-β, CXCL9, CXCL10 and S100A8/A9 were higher in those who later experienced disease relapse (Fig. 1C, Table 2). For the median values of S100A8/A9 (p = 0.016) and CXCL9 (p = 0.031) the statistical difference was confirmed in post-hoc Bonferroni corrections for multiple comparisons (Table 2).

**Table 2:** Performance of biomarkers showing significant differences (all in pg/ml except IFNβ in U/ml and S100A8/A9 in ng/ml).

Marker	IBD (n = 40)					UC (n = 30)				
	Stable Remission Median (95%CI)	Unstable Remission Median (95%CI)	AUC (95%CI)	P^a^	P adj.^b^	Stable Remission Median (95%CI)	Unstable Remission Median (95%CI)	AUC (95%CI)	P^a^	P adj.^b^
IL-1Ra	280 (188–651)	511 (257–955)	0.604 (0.42–0.78)	0.222	n.s.	278 (151–438)	515 (423–955)	0.72 (0.53–0.91)	**0.040**	n.s.
IL-1β	1.83 (0.8–2.25)	3.6 (1.03–8.63)	0.706 (0.53–0.87)	**0.030**	n.s.	1.79 (0.4–2.25)	3.62 (1.03–8.63)	0.742 (0.56–0.92)	**0.023**	n.s.
IL-2	1.07 (0.1–2.93)	0.1 (0.1–4.47)	0.601 (0.42–0.78)	0.253	n.s.	0.92 (0.1–2.93)	0.1 (0.1–6.23)	0.515 (0.30–0.73)	0.886	n.s.
IL-8	18.16 (12.51–34.72)	26.47 (18.65–68.36)	0.643 (0.46–0.82)	0.182	n.s.	13.80 12.06–24.72)	28.06 (20.60–68.36)	0.769 (0.59–0.94)	**0.011**	n.s.
IL-13	6.51 (4.38–10.16)	13.12 (8.37–29.69)	0.678 (0.50–0.85)	0.050	n.s.	5.31 (1.25–15.08)	16.64 (9.12–29.69)	0.733 (0.55–0.92)	**0.029**	n.s.
IL-15	4.41 (3.26–6.43)	7.46 (4.0–9.4)	0.69 (0.53–0.86)	**0.038**	n.s.	4.57 (3.26–7.75)	8.17 (4.91–9.40)	0.729 (0.54–0.91)	**0.033**	n.s.
IL-18	85.36 (67.81–142.2)	136.5 (118.9–166.3)	0.726 (0.56- 0.89)	**0.015**	n.s.	119 (66.34–156)	131 (119–156)	0.644 (0.44–0.85)	0.187	n.s.
IL-21	884 (126–1451)	1779 (682–6266)	0.722 (0.55–0.89)	**0.030**	n.s.	898 (10–1451)	2182 (1326–6266)	0.782 (0.61–0.95)	**0.007**	**0.038**
IL-25	1132 (329–1580)	2422 (617–6174)	0.738 (0.57–0.90)	**0.010**	n.s.	1098 (28.28–1687)	2847 (617–8210)	0.778 (0.61–0.95)	**0.009**	n.s.
IFNβ	868 (10–1260)	1713 (10–4492)	0.692 (0.52- 0.86)	**0.037**	n.s.	137 (10–1260)	2501 (10–4492)	0.733 (0.54–0.92)	**0.026**	n.s.
CXCL9	28.98 (16.74–62.15)	116.5 (30.87–116.5)	0.746 (0.59- 0.89)	**0.007**	**0.031**	27.36 (13.51–37.82)	62.97 (35.6–116.5)	0.804 (0.64–0.96)	**0.004**	**0.012**
CXCL10	211 (159–259)	268 (238–597)	0.692 (0.52- 0.86)	**0.041**	n.s.	209 (129–252)	322 (238–597)	0.796 (0.63–0.96)	**0.005**	**0.024**
CXCL11	91 (25.4–109.8)	141 (67.4–191.5)	0.700 (0.53 0.87)	0.069	n.s.	35.31 (22.49–98.95)	151 (46.53–192)	0.787 (0.61–0.96)	**0.007**	**0.008**
G-CSF	16.45 (89.6–20.86)	53.69 (14.28–193.7)	0.670 (0.49- 0.85)	0.054	n.s.	17.57 (9.6–20.86)	61.4 (20.1–194)	0.769 (0.58–0.95)	**0.011**	n.s.
Galectin-1	37,870 (29,212–44,078)	46,047 (30,311–59,136)	0.658 (0.48–0.83)	0.095	n.s.	38,373 (35,492–46,523)	46,422 (38,745–60,042)	0.752 (0.57–0.93)	**0.011**	**0.002**
S100A8/A9	2040 (1000–3100)	3070 (2600–4500)	0.757 (0.60- 0.91)	**0.005**	**0.016**	2160 (1450–3200)	3920 (3270–6100)	0.849 (0.69–0.99)	**0.001**	**< 0.001**

*CI* confidence interval, *AUC* area under curve; ^a^significance of ROC analyses; ^b^adjusted significance using Kruskal Wallis test with Bonferroni post-hoc correction for multiple comparisons.

Significant P-values indicated in bold.

Standard laboratory markers (Hb, white blood cell counts, and CRP; Table 1) in the group with future IBD relapse did not significantly differ from those in the future non-relapse group. In addition, fecal markers of inflammation (fecal calprotectin and S100A12, respectively) revealed no significant differences between the patient groups (Supplementary Table S1). In contrast to the differences in baseline levels and except for CXCL10, there was no clear trend when comparing the T1 with T2 samples. The differences between inactive and active disease appear rather small in comparison with the differences observed with background activity at inclusion (Fig. 1C).

In patients with unstable remission, experiencing a future flare, multiple correlation analyses of significantly different serum markers in IBD T1-samples (Fig. 1C, Table 2) as well as routine blood and fecal markers of inflammation (Table 1; Supplementary Table S1) revealed that mainly markers which can be linked to T cell activation (IL-15, IL-18, IL-21, IL-25) or IFNγ-signaling (CXCL9) but also IL-1β and IFNβ cluster together in positive association (Fig. 2). This pattern only marginally differs from that observed in patients remaining in stable remission. Serum and inflammatory marker associations in these patients with stable remission predominantly reveal marked negative correlations with fecal calprotectin, which is inverse to what we observed with respect to fecal S100A12 (Fig. 2).

Restricting our analyses to UC patients (Fig. 3A) did not benefit the overall unsupervised clustering based on the acquired levels of 52 serum markers (Fig. 3B), but we observed that concentrations of 14 analytes (IL-1β, IL-1RA, IL-8, IL-13, IL-15, IL-21, IL-25, IFN-β, CXCL9, CXCL10, CXCL11, S100A8/A9, G-CSF and Galectin-1) were significantly higher in samples from patients with unstable remission compared to patients with stable remission (Fig. 3C, Table 2). For the median values of S100A8/A9 (p < 0.001), Galectin-1 (p = 0.002), CXCL11 (p = 0.008), CXCL9 (p = 0.012), CXCL10 (p = 0.024), and IL-21 (p = 0.038) the statistical difference was confirmed in post-hoc Bonferroni corrections for multiple comparisons (Table 2).

Paralleling observations in the total IBD cohort, standard blood or fecal markers of inflammation (Hb, white blood cell counts, CRP, fecal calprotectin, fecal S100A12) in patients with future relapse did not significantly differ from those with future stable remission (Table 1, Supplementary Table S1). When comparing biomarker levels in T1 with T2 samples we only observed significant differences in CXCL10 and CXCL11 (Fig. 3C). Most patients (n = 27) within the UC cohort were seen within less than 60 days following T1 for their respective follow-up T2 visit. When excluding three patients with T2 visits > 100 days from our data set, this did not result in major changes among the identified markers with significantly different serum levels with respect to future flare or stable remission (Supplementary Table S2).

When subjecting only data acquired from UC patients to multiple correlation analyses, the picture marginally differs from what we observed in total IBD. Both among patients with future relapse or stable remission we observed mainly markers linked to T cell activation (IL-15, IL-18, IL-21, IL-25) or IFNγ-signaling (CXCL9, CXCL11) but also IL-1β and IFNβ to cluster together in positive association (Fig. 4). Associations of serum cytokines with IL-8, G-CSF, CXCL10, S100A8/A9 and Galectin-1 appear to mainly differ in T1-samples obtained from patients remaining in remission or experiencing a future flare. Further, as observed in the total IBD cohort, serum and inflammatory marker associations in patients with future stable remission predominantly reveal marked negative correlations with fecal calprotectin, which is inverse to what we observed with respect to fecal S100A12 (Fig. 4).

Prompted by ROC analyses revealing differences between patients with future stable versus unstable remission for both the whole IBD and the UC cohort (Table 2), we evaluated predictive models taking the significant biomarkers into account. The analyses have to be interpreted with care due to the balance of patient samples and analytes and the limitations of multiple comparisons. Binary nominal logistic regression analyses showed that S100A8/A9 has a predictive power for all IBD patients and even better for UC patients. In the latter group, a model adding S100A8/A9 to measurements of CXCL11 yields a predictive power of 80% (Supplementary Tables S2 and S3). In UC patients, both CXCL11 (sensitivity 67%, specificity 87%, likelihood ration 5.0) and S100A8/A9 (sensitivity 73%, specificity 87%, likelihood ration 5.5) could be confirmed as markers differentiating patients with future flares from those with stable remission (Supplementary Table S4).

## Discussion

While it is unlikely that serum biomarkers will ever replace invasive tests, such as endoscopy, they could be useful as inflammatory markers filtering for the need of invasive investigations while monitoring the patients’ disease course. We extend our previous work that has revealed a predictive power of fecal S100A12 and calprotectin, but only a weak association of their serum levels with flare risk in IBD patients. For further analyses of candidate blood-based markers, we created a cohort of patients who either remained in remission during follow up (“stable remission” group) or who consecutively experienced a relapse in a predefined time period (“unstable remission” group). We present 16 biomarkers with the potential to indicate unstable remission in IBD. In particular, 14 molecular markers with elevated values in UC patients with unstable remission were identified. Those can be indicative of a background T(h1) cell activation, but also innate immune activation (e.g., shown by S100A8/A9) that is more pronounced in those considered in clinical remission who likely continue having subclinical inflammatory processes.

There were only relatively small alterations in the biomarker concentrations when comparing T1 and T2 samples, both in patients with stable and unstable remission. It appears conceivable that the fluctuations in serum biomarkers during inactive and active disease phases in individual patients are less prominent than dysbalanced immune activity that is present as a background characteristic of the patient group, as these differences are even observed during times of clinically inactive disease in remission. Although our results have to be interpreted with caution in light of the limited patient numbers, the correlograms indicate that differences in T cell activation and to some extent also innate immunity as well as IFN-related pathways may influence a background immune activation that can influence the risk of relapsing disease.

A set of markers used to indicate molecular signatures may be more suitable for precision medicine than single biomarkers, as they can unmask complex processes rather than a single phenomenon. For patients with Rheumatoid Arthritis (RA), treat-to-target strategies and tight control are nowadays cornerstones of patient management, and biomarker panels have been introduced and validated for clinical purposes^32^. There are important distinctions between RA, CD and UC, suggesting differences in the underlying pathways driving each disease. However, the unifying treatment target is disease remission, mainly defined as clinical remission supported by endoscopy or imaging. Biomarker remission (normal blood cells counts, CRP, fecal calprotectin) is considered as an adjunctive target^3^. Although fecal markers are considered non-invasive means to monitor intestinal inflammation, the lack of conclusive data on relapse prediction and the low acceptance of stool sampling by patients is limiting their widespread use^19–23^. A need therefore exists for blood-based biomarkers that accurately detect disease activity in IBD.

A recent study by D’Haens et al. used a commercial assay of multiple markers indicating mucosal damage and repair processes (PROMETHEUS Monitr Crohn’s Disease Test) to calculate an endoscopic healing index (EHI) that identifies patients with resolution of endoscopic disease activity^33^. The test applies a proprietary algorithm with 13 biomarkers to produce a quantitative EHI score. The authors used very strong outcome measures of endoscopic healing that were not available to us. The study also didn’t test the prediction of remission of risk or relapses. Some of the biomarkers that revealed promising results in our small IBD cohort were not considered by D’Haens et al., because they showed poor analytical reproducibility, low detection rate, and/or lack of correlation to disease severity in preliminary studies. These markers were eliminated from further consideration, and data are not presented. The study was funded by Prometheus, several authors were either employees or at least had a relation to Prometheus, and the company was responsible for running assays and analyses that are only partially reported^34^. Even though this limits the interpretation of data, it appears promising that the accuracy of the blood-based multi-marker set was comparable to fecal calprotectin and better than measurement of serum CRP.

As a limitation, we could only use clinical disease activity indices (CDAI and UCAI) to define stable and unstable remission. Endoscopic or histological disease activity measures were not available from the participants included in remission. It is a drawback of CDAI/UCAI scores that they may not correlate well with endoscopically proven intestinal inflammation. The relatively small sample number limits the statistical power of our study. Especially the number of samples from CD patients available for the retrospective project is a significant limitation. This also excluded further stratification of patients, e.g. with regard to disease characteristics or therapies. As an example, two CD patients received systemic steroids at inclusion, one reduced the dose at visit 1 from 5 to 4 mg/day, and the other one actually stopped as visit 1. We cannot fully exclude that medication changes in therapy influence the risk of flare at future time point due to poorly controlled disease. In addition, we could not correlate our data to repeated endoscopic measures in the cohort recruited in disease remission. However, we consider the results promising and in line with other studies showing that serum biomarker panels have a potential to identify IBD from symptomatic controls and to predict future disease course^35^. Future studies will need to confirm whether elevated inflammatory markers in IBD patients in clinical remission as defined by clinical disease activity indices may represent a stage of residual inflammation, which progresses to cause an eventual clinical relapse of the disease. Conversely, it is conceivable that measuring biomarker panels may serve as a tool for measuring the effects of treatment. Consequently, treatment of IBD could be tapered at a point where the biomarkers suggest that the relapse of disease is unlikely to occur within a defined period. Our analyses indicate that especially S100A8/A9 analyses may have a predictive power for all IBD patients and even better for UC patients. A recent other study also suggested S100A8/A9 and either CRP or albumin for a prognostic model to predict treatment escalation in IBD^36^.

In conclusion, there is a strong need for defining appropriate variables for follow-up recommendations which is vital to IBD treatment and management. Based on our results, it seems a feasible goal to apply molecular signatures, measurable in blood and available for tight monitoring of disease activity. Future studies will test the treat-to-target approaches in larger cohorts and can be used to validate the usefulness of biomarker signatures for tight monitoring of disease activity.

## Supplementary Information

Supplementary Tables.
