# Supplementary material for: Serum biomarkers confirming stable remission in inflammatory bowel disease
Source: Sci Rep. 2021 Mar 23;11:6690. doi: 10.1038/s41598-021-86251-w (PMC7988138; doi:10.1038/s41598-021-86251-w)
Supplement: Supplementary file 1 — Supplementary Tables. [file 41598_2021_86251_MOESM1_ESM.pdf]

Supplementary Material

Serum biomarkers confirming stable remission in inflammatory bowel disease

Christoph Kessel, PhD[1,*], Miha Lavric, PhD[1,2,*], Toni Weinhage, PhD[1], Markus Brueckner, MD[3], Sytze de Roock, MD[4], Jan Däbritz, MD[5], Jakob Weber, PhD[6], Sebastiaan J. Vastert, MD[4], Dirk Foell, MD[1]

*[1]Pediatric Rheumatology and Immunology, University Hospital Muenster, Germany; [2]Research Group Ambient Intelligence, Saxion University of Applied Sciences, Enschede, Netherlands; [3]Department of Gastroenterology and Hepatology, University Hospital Muenster, Germany; [4]Department of Pediatric Rheumatology and Immunology and Center for Translational Immunology, University Medical Center Utrecht, Netherlands; [5]Department of Pediatrics, University Medical Center Rostock, Germany; [6]BÜHLMANN Laboratories AG, Schoenenbuch, Switzerland; *authors contributed equally*

SUPPLEMENTARY TABLE S1. Comparison of fecal biomarkers (in mg/kg)

	IBD (n=40)				UC (n=30)			
**Marker**	**Stable Remission Median (95%CI)**	**Unstable Remission Median (95%CI)**	**AUC (95%CI)**	**P[a]**	**Stable Remission Median (95%CI)**	**Unstable Remission Median (95%CI)**	**AUC (95%CI)**	**P[a]**
**fecal S100A12**	0.90 (0.09-15.50)	1.20 (0.04-11.70)	0.558 (0.36-0.75)	0.567	0.86 (0.09-4.15)	1.25 (0.11-11.70)	0.661 (0.45-0.87)	0.169
**Fecal Calprotectin**	30.4 (1.3-365.5)	62.1 (13.2-350.0)	0.674 (0.49- 0.86)	0.089	25.9 (13.2-128.5)	54.5 (1.5-365.5)	0.591 (0.38-0.80)	0.395

CI, confidence interval; AUC, area under curve; [a] significance of ROC analyses

SUPPLEMENTARY TABLE S2. Performance of significant serum biomarkers in UC (T2<60 days, n=27) with significant differences at T1

UC (with T2 in < 60 days, n=27)					
**Marker**	**Stable Remission Median (95%CI)**	**Unstable Remission Median (95%CI)**	**AUC (95%CI)**	**P[a]**	***P adj.*[b]**
**IL-1β**	1.38 (0.4-2.25)	3.62 (1.03-8.63)	0.761 (0.58-0.94)	**0.022**	*n.s.*
**IL-8**	15.62 12.51-43.53)	28.06 (20.60-68.36)	0.739 (0.54-0.94)	**0.036**	*n.s.*
**IL-15**	3.99 (2.99-5.63)	8.17 (4.91-9.40)	0.800 (0.63-0.97)	**0.008**	***0.045***
**IL-21**	925 (10-2857)	2182 (1326-6266)	0.767 (0.59-0.95)	**0.019**	*n.s.*
**IL-25**	962 (28.28-1683)	2847 (617-8210)	0.789 (0.62-0.96)	**0.011**	*n.s.*
**IFNβ**	73.41 (10-1260)	2501 (10-4492)	0.756 (0.57-0.94)	**0.025**	*n.s.*
**CXCL9**	26.32 (13.51-42.15)	62.97 (35.6-116.5)	0.794 (0.61-0.97)	**0.009**	***0.040***
**CXCL10**	212 (124-277)	322 (238-597)	0.783 (0.61-0.96)	**0.013**	***0.017***
**CXCL11**	26.6 (12.05-84.27)	151 (46.53-192)	0.822 (0.66-0.99)	**0.005**	***0.007***
**G-CSF**	15.99 (9.6-27.00)	61.4 (20.1-194)	0.756 (0.56-0.95)	**0.025**	*n.s.*
**S100A8/A9**	2215 (1480-3300)	3920 (3270-6100)	0.822 (0.64-1.00)	**0.005**	***0.017***

All in pg/ml, except for S100A8/A9 (ng/ml) and IFNβ (U/ml); CI, confidence interval; AUC, area under curve; [a] significance of ROC analyses; [b] adjusted significance using Kruskal Wallis test with Bonferroni post-hoc correction for multiple comparisons

SUPPLEMENTARY TABLE S3. IBD relapse prediction model according to binary logistic regression

Step 0			Step 1		
**Variable**	**Score**	**P**	**Change in -2 Log Likelihood**	**P**	**Model correct prediction (%)**
**S100A8/A9**	**5.548**	**0.019**	**5.883**	**0.015**	**70**
**CXCL9**	**5.312**	**0.021**	2.898	0.089	
**CXCL10**	**4.011**	**0.045**	2.774	0.096	
CXCL11	2.720	0.099	2.718	0.099	
IL-21	2.064	0.151	2.207	0.137	
Galectin-1	1.630	0.202	0.300	0.584	

SUPPLEMENTARY TABLE S4. UC relapse prediction model according to binary logistic regression

Step 0			Step 1			Step 2		
**Variable**	**Score**	**P**	**Change in -2 Log Likelihood**	**P**	**Model correct prediction (%)**	**Change in -2 Log Likelihood**	**P**	**Model correct prediction (%)**
**CXCL11**	**8.373**	**0.004**	**9.563**	**0.002**	**67**	**5.956**	**0.015**	**80**
**S100A8/A9**	**8.061**	**0.005**	**5.211**	**0.022**	**-**	**5.509**	**0.019**	**80**
**CXCL9**	**5.908**	**0.015**	1.570	0.210		0.155	0.693	
**IL-21**	**5.638**	**0.018**	2.048	0.152		1.483	0.223	
**CXCL10**	**5.166**	**0.023**	2.050	0.152		3.121	0.077	
Galectin-1	2.830	0.093	2.071	0.150		2.072	0.150	

SUPPLEMENTARY TABLE S5. Performance of markers with predictive potential

	IBD (n=40)					UC (n=30)				
**Marker**	**AUC (95%CI)**	**Cut-Off**	**Sensitivity**	**Specificity**	**LR**	**AUC (95%CI)**	**Cut-Off**	**Sensitivity**	**Specificity**	**LR**
**CXCL11**	0.700 (0.53-0.87)	131 pg/ml	58%	85%	3.5	0.787 (0.61-0.96)	102 pg/ml	67%	87%	5.0
**S100A8/A9**	0.757 (0.60-0.91)	3205 ng/ml	70%	80%	3.5	0.849 (0.69-0.99)	3475 ng/ml	73%	87%	5.5

CI, confidence interval; AUC, area under curve; LR, likelihood ratio
